# Supplementary material for: Region-specific and state-dependent action of striatal GABAergic interneurons
Source: Nat Commun. 2018 Aug 21;9:3339. doi: 10.1038/s41467-018-05847-5 (PMC6104028; doi:10.1038/s41467-018-05847-5)
Supplement: Supplementary file 1 — Supplementary Information [file 41467_2018_5847_MOESM1_ESM.pdf]

Supplementary Information

Region-specific and state-dependent action of striatal GABAergic interneurons

Fino E, Vandecasteele M, Perez S, Saudou F and Venance L

Contents

Supplementary Figures 1-12

Supplementary Tables 1-2

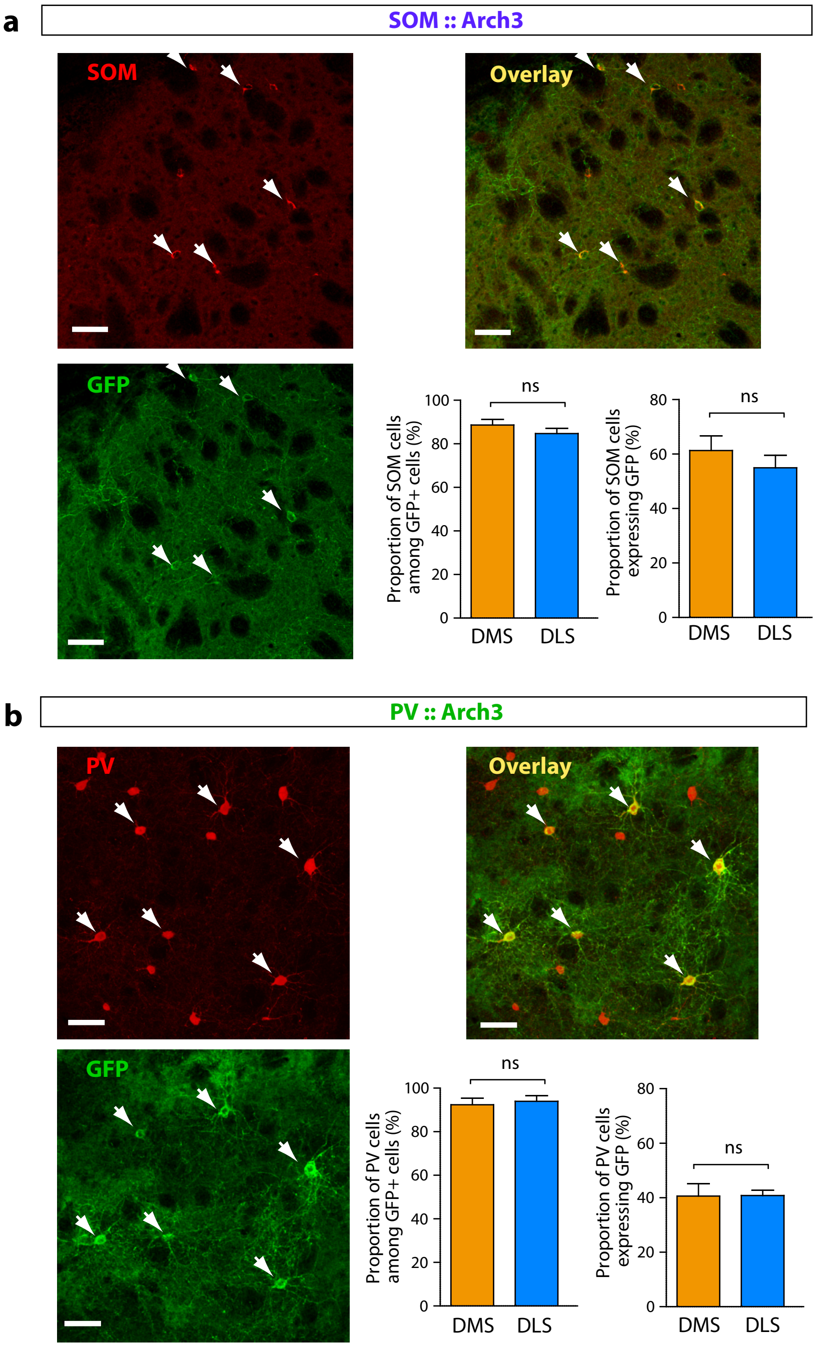

Supplementary Figure 1: Specific expression of Arch3 in PV and SOM cells

(**a**) Confocal microscopy image of immunostainings for SOM and GFP in SOM::Arch3-GFP mice. Arrows indicate cells co-expressing SOM and GFP (scale bar: 50 µm). Bar graphs show quantification of the co-expression and indicate a specific expression of Arch3-GFP in SOM cells since in both DMS and DLS, nearly all the GFP+ cells are SOM+ cells. In addition, in these transgenic mice, the expression of Arch3-GFP is effective in about 55% of SOM cells with no differences between DMS and DLS (61.29±5.40% in DMS and 54.93±4.63% in DLS, n= 6 mice, p=0.38). (**b**) Confocal microscopy image of immunostainings for PV and GFP in PV::Arch3-GFP mice. Arrows indicate cells co-expressing PV and GFP (scale bar: 50 µm). Bar graphs show quantification of the co-expression and indicate a specific expression of Arch3-GFP in PV cells since in both DMS and DLS, nearly all the GFP+ cells are PV+ cells. In addition, in these transgenic mice, the expression of Arch3-GFP is effective in about 40% of PV cells with no differences between DMS and DLS (40.61±4.54% in DMS and 40.77±1.95% in DLS, n= 3 mice, p=0.95).

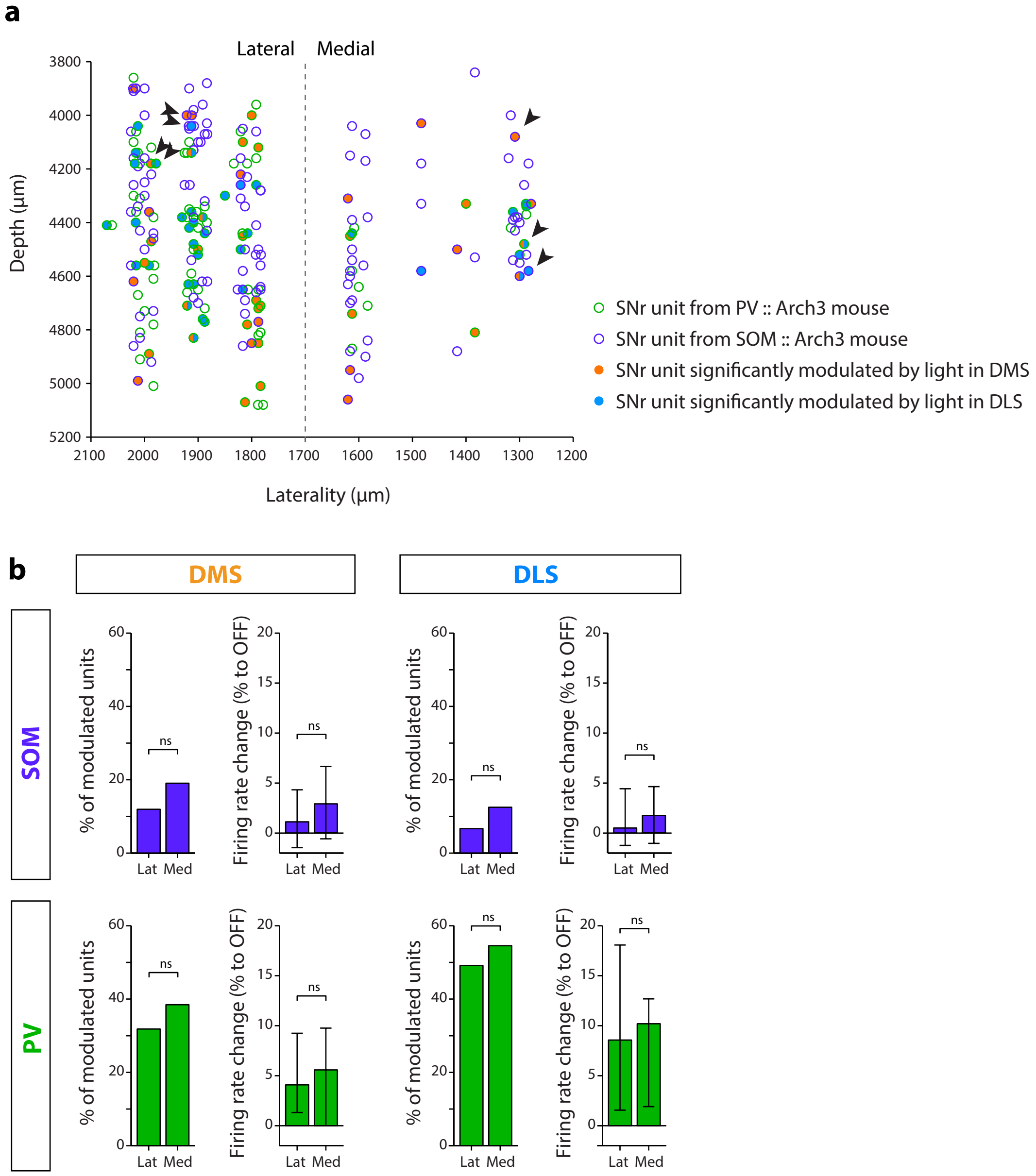

Supplementary Figure 2: Absence of relationship between the localization of recorded SNr units and the effect of opto-inhibition of striatal interneurons on SNr activity.

(**a**) Recording coordinates of all SNr units from SOM::Arch3 (purple circles) and PV::Arch3 (green circles). Filled colors indicate units that were individually significantly modulated by opto-inhibition of either SOM or PV interneurons in DMS (orange) and/or in DLS (blue). Arrowheads illustrate that for each of the 4 conditions (SOM-DMS, PV-DMS, SOM-DLS, PV-DLS), significantly modulated units were recorded in both medial and lateral locations. (**b**) The effect of opto-inhibition of SOM or PV striatal interneurons in DMS or DLS did not depend on the laterality of recorded units. We divided the recorded units in a more lateral and more medial part (dashed line in (a)), and quantified for each condition the proportion of significantly modulated units and the median change on the firing frequency separately for medially and laterally recorded units. In all conditions, neither the proportion nor the effect were significantly different depending on the laterality (proportions : fisher's exact test SOM-DMS p=0.4053, PV-DMS p=0.749, SOM-DLS p=0.4124, PV-DLS p=1; firing rate change: Mann-Whitney-Wilcoxon test SOM-DMS p=0.0996, PV-DMS p=0.9420, SOM-DLS p=0.7195, PV-DLS p=0.7899). For each condition, there was no significant correlation between the effect of opto-inhibition of striatal interneurons (median change in firing rate) and either the depth or the laterality of the recording site (spearman correlations: SOM-DMS with laterality r=-0.1340, p=0.1647, with depth r=0.1547, p=0.1082, n=109 units; PV-DMS with laterality r=-0.0933, p=0.4135, with depth r=0.0942, p=0.4089, n=79 units; SOM-DLS with laterality r=0.006, p=0.9963, with depth r=0.2233, p=0.0651, n=68 units; PV-DLS with laterality r=0.2073, p=0.0898, with depth r=0.1708, p=0.1637, n=68 units).

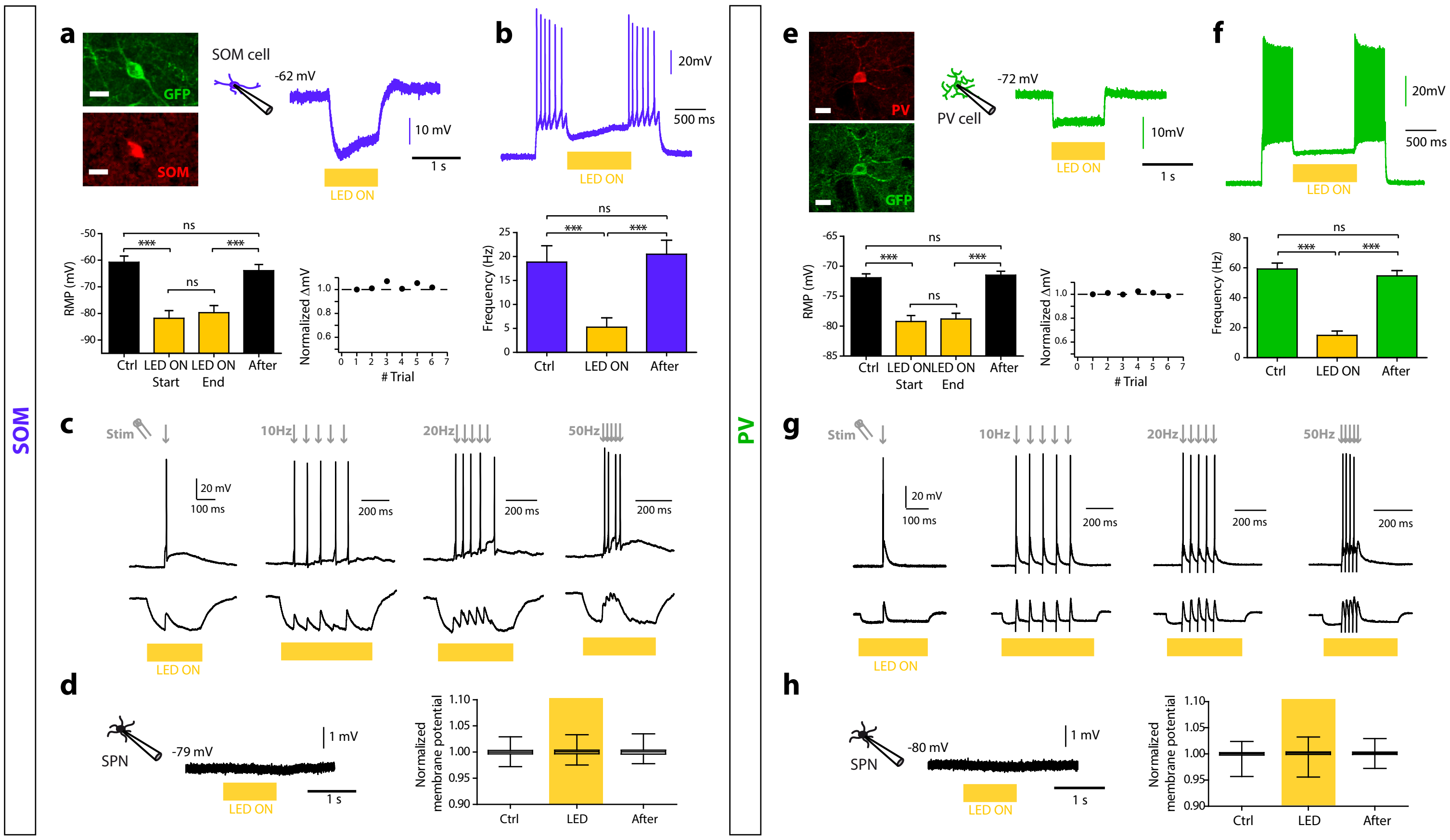

Supplementary Figure 3: Reliable opto-inhibition of PV and SOM interneurons expressing Arch3.
(**a**) Confocal images of a striatal SOM interneuron with expressing Arch3-GFP and the same one with SOM immunostaining (scale bar: 20 µm). Representative responses of a SOM interneuron to photostimulation with yellow light (1 sec light pulses). Yellow light pulses induce strong hyperpolarization in SOM cells (n=16). The averaged light-induced hyperpolarization was 24.2±3.0 mV and was efficient, stable and reproducible. (**b**) Representative trace and summary bar graph showing that Arch3 activation efficiently blocks APs evoked in SOM cells by a depolarizing current step (averaged frequency in control 18.9±3.4 Hz vs during LED ON 5.3±2.0 Hz, n=16, p<0.0001, ratio: 0.19±0.04). (**c**) Arch3 expression in SOM cells also allows blocking cortically-evoked APs in all tested neurons (n=8). The effect is reliable and effective for single stimulation but also for different frequencies of trains of cortical stimulation (10, 20, 50 Hz). Yellow bars illustrate the opto-inhibition period. (**d**) Representative responses of a SPN to photostimulation (1 sec LED) showing that LED has no effect on surrounding SPNs (n=27). Whisker Boxes showing the normalized membrane potential before, during and after the LED pulse. There is no significant difference between all the conditions ($F_{2,382}$=0.3635, p=0.6955, One-way Anova). (**e**) Confocal microscopy image of a striatal PV interneuron expressing Arch3-GFP and the same one with PV immunostaining (scale bar: 20 µm). Representative responses of a PV interneuron to photostimulation with yellow light (1 sec light pulse). Yellow light pulses induce strong hyperpolarization in PV cells (n=12). The averaged light-induced hyperpolarization is 9.5±0.8 mV and the hyperpolarization was efficient, stable and reproducible. (**f**) Representative trace and summary bar graph showing that Arch3 activation efficiently blocks APs evoked in PV cells by a depolarizing current step (averaged frequency in control 59.2±4.0 Hz vs during LED ON 14.9±2.9 Hz, n=12, p<0.0001, ratio: 0.25±0.04). (**g**) Arch3 expression in PV cells also allows to block cortically-evoked APs in all tested neurons (n=11). The effect is reliable and effective for single stimulation but also for different frequencies of cortical stimulation trains (10, 20, 50 Hz). (**h**) Representative responses of a SPN to photostimulation (1 sec LED) showing that LED has no effect on surrounding SPNs (n=28). Whiskers Boxes showing the normalized membrane potential before, during and after the LED pulse. There is no significant difference between all the conditions ($F_{2,387}$=1.255, p=0.2862) * p<0.05, ** p<0.01, *** p<0.001.

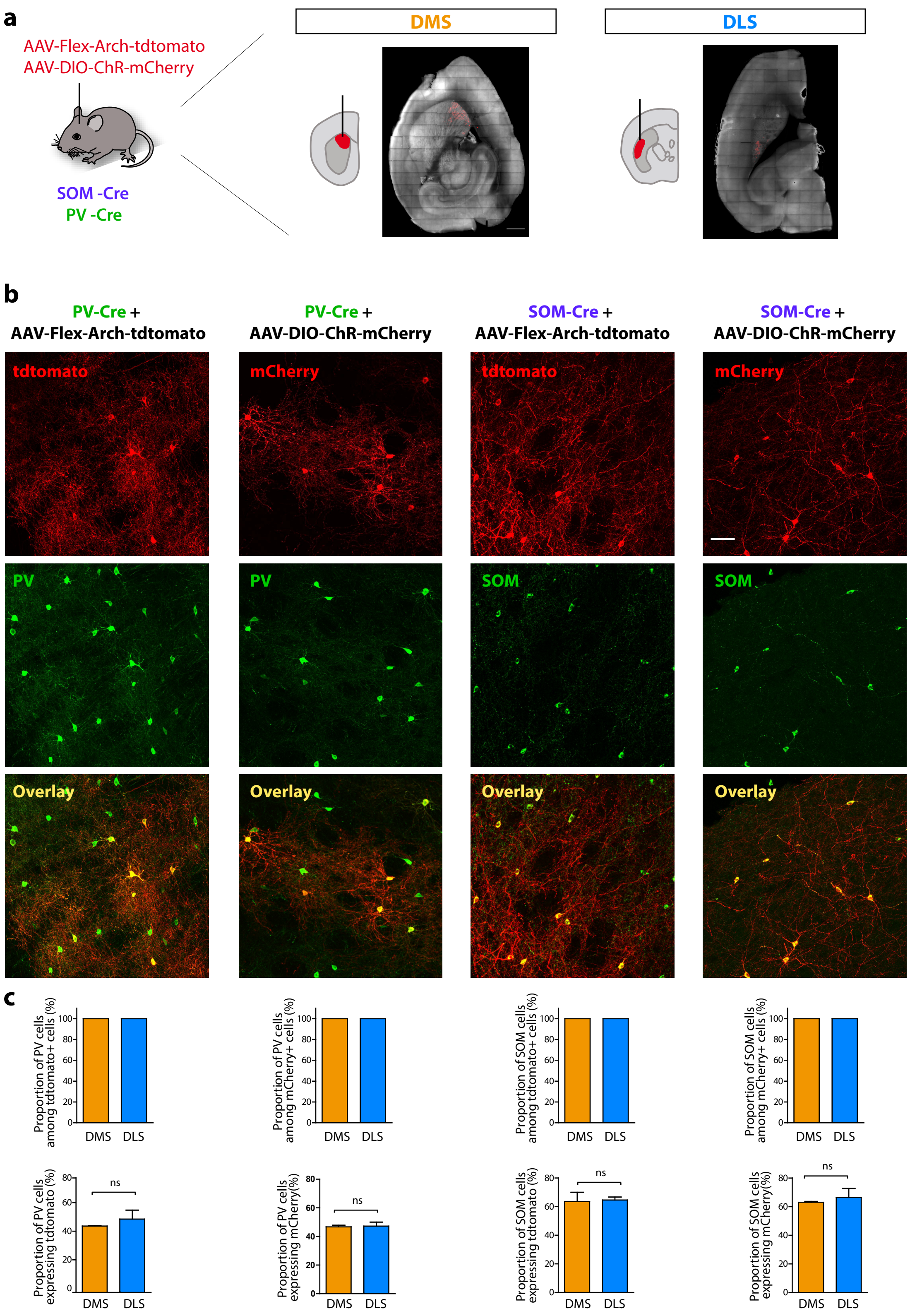

Supplementary Figure 4: Specific expression of ArchT and ChR2 in PV and SOM cells after viral injections. (**a**) Illustration of the injections sites of AAV vectors in the DMS (shown on a para-sagittal slice) or DLS (shown on a horizontal slice). (**b**) Confocal microscopy image of immunostainings for PV/SOM and tdtomato/mCherry two weeks after the injections (scale bar: 50 µm). (**c**) Bar graphs show quantification of the co-expression of PV and SOM with the virally delivered opsins. There is a specific expression of ArchT-tdtomato and ChR2-mCherry in PV or SOM cells since in both DMS and DLS, 100% of the tdtomato+ or mCherry+ cells are PV+ or SOM+ cells. Similarly to transgenic mice, the expression of ArchT-tdtomato and ChR2-mCherry is effective in about 40% of PV cells with no differences between DMS and DLS (46.41±0.26% for ArchT-tdtomato and 46.64±1.18% for ChR2-mCherry in DMS and 51.25±6.25% for Arch-tdtomato and 47.22±2.78% for ChR-mCherry in DLS, p=0.52 for Arch-tdtomato and p=0.8650 for ChR-mCherry, n=2 mice for each condition). Concerning SOM cells, similarly to transgenic mice, the expression of Arch-tdtomato and ChR-mCherry is effective in about 60% of SOM cells with no differences between DMS and DLS (63.57±6.43% for Arch-tdtomato and 63.07±0.57 % for ChR-mCherry in DMS and 64.58±2.08% for Arch-tdtomato and 66.36±6.36 % for ChR-mCherry in DLS, p=0.895 for Arch-tdtomato and p=0.6574 for ChR-mCherry, n=2 mice for each condition).

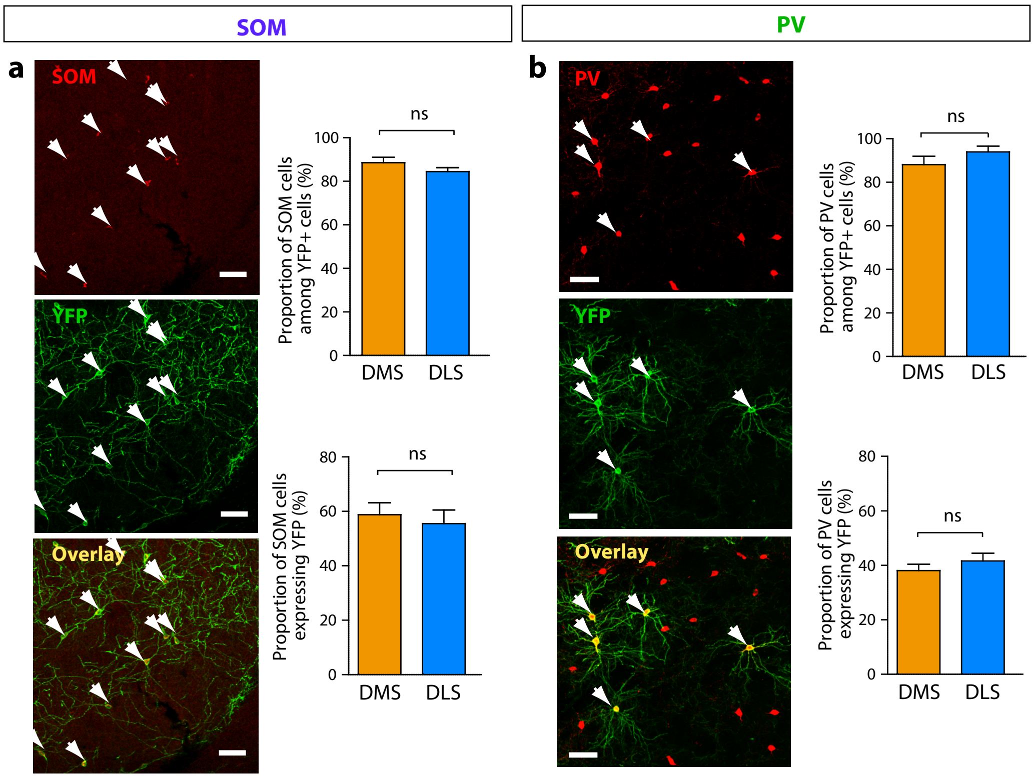

Supplementary Figure 5: Specific expression of ChR2 in PV and SOM cells

(**a**) Confocal microscopy image of immunostainings for SOM and YFP in SOM-ChR2-YFP mice. Arrows indicate cells co-expressing SOM and YFP (scale bar: 50 µm). Bar graphs show quantification of the co-expression and indicate a specific expression of ChR2-YFP in SOM cells since in both DMS and DLS, nearly all the YFP+ cells are SOM+ cells. In addition, in these transgenic mice, the expression of ChR2-YFP was effective in about 55% of SOM cells with no differences between DMS and DLS (58.76±4.38% in DMS and 55.53±4.94% in DLS, n=5 mice, p=0.63). (**b**) Confocal microscopy image of immunostainings for PV and YFP in PV-ChR2-YFP mice. Arrows indicate cells co-expressing PV and YFP (scale bar: 50 µm). Bar graphs show quantification of the co-expression and indicate a specific expression of ChR2-YFP in PV cells since in both DMS and DLS, nearly all the YFP+ cells were PV+ cells. In addition, in these transgenic mice, the expression of ChR2-YFP was effective in about 40% of PV cells with no differences between DMS and DLS (38.1±2.3% in DMS and 41.6±2.9% in DLS, n= 3 mice, p=0.37).

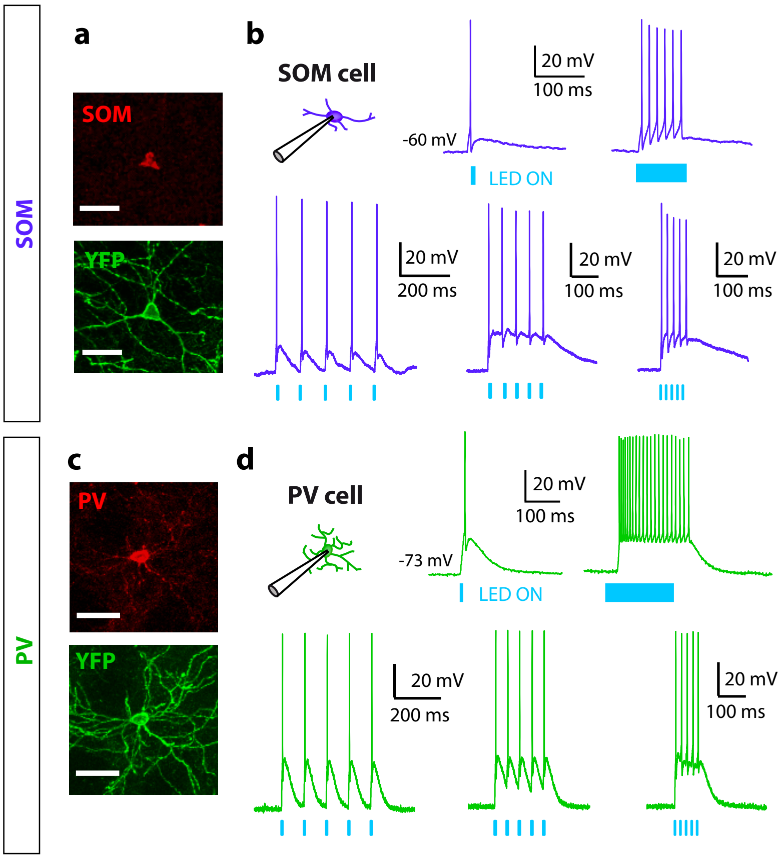

Supplementary Figure 6: Reliable opto-activation of SOM and PV interneurons expressing ChR2.
(**a**) Magnification of an interneuron expressing ChR2-YFP and SOM (scale bar: 50 µm). (**b**) In all of the SOM cells recorded (n=7, one representative shown), we were able to evoke APs with 5ms light stimulation with 100% success rate, at all tested frequencies and both in DMS or DLS. A 300 ms optical stimulation induced 13.25±0.94 APs and 12.67±1.20 APs in DMS (p=0.7143, n=7 SOM cells). Top panels: APs evoked in SOM interneurons by opto-stimulation with blue light (5 ms or 100 ms light pulses). Lower panels: trains of blue light pulses (5 ms) at various frequencies (10, 20 and 50 Hz) led to SOM interneurons firing. Blue bars illustrate the light stimulation period. (**c**) Magnification of an interneuron expressing ChR2-YFP and PV (scale bar: 50 µm). (**d**) In all of the PV cells recorded (n=8, one representative shown), we were able to evoke APs with each 5ms light stimulation with 100% success rate, at all tested frequencies and both in DMS or DLS. A 300ms optical stimulation induced 14.30±0.68 APs and 14.85±2.28 APs in DMS (p=0.8283, n=8 PV cells). Top panels: APs evoked in PV interneurons by photostimulation with blue light (5 ms or 200 ms light pulses). Lower panels: trains of blue light pulses (5ms) at various frequencies (10, 20 and 50 Hz) reliably drive PV interneurons to evoke APs.

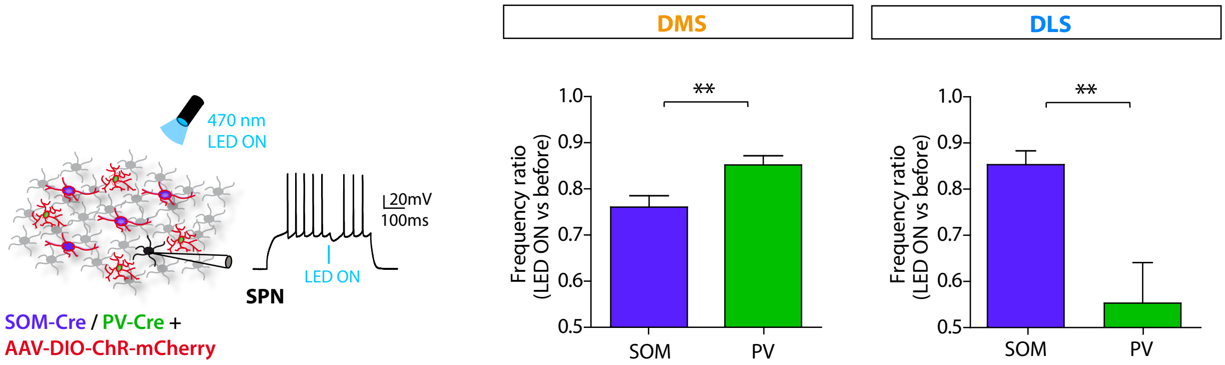

Supplementary Figure 7: Differential effect of PV and SOM cells on firing SPNs after viral injections. After AAVs injection, we measured the effect of short opto-activation (5ms, 470 nm excitation LED) of PV and SOM cells on SPN frequency discharge induced by depolarizing current steps (500ms). Right, Frequency inhibition ratio (frequency during LED ON vs before LED ON) in SPNs induced by 5ms light pulse. Frequency decrease was significantly higher for SOM cells (purple) activation in DMS territory (p=0.0099, n=7 SOM cells and n=6 PV cells) while this decrease was significantly higher for PV cells (green) activation in DLS territory (p=0.0021, n=5 SOM cells and n=7 PV cells).

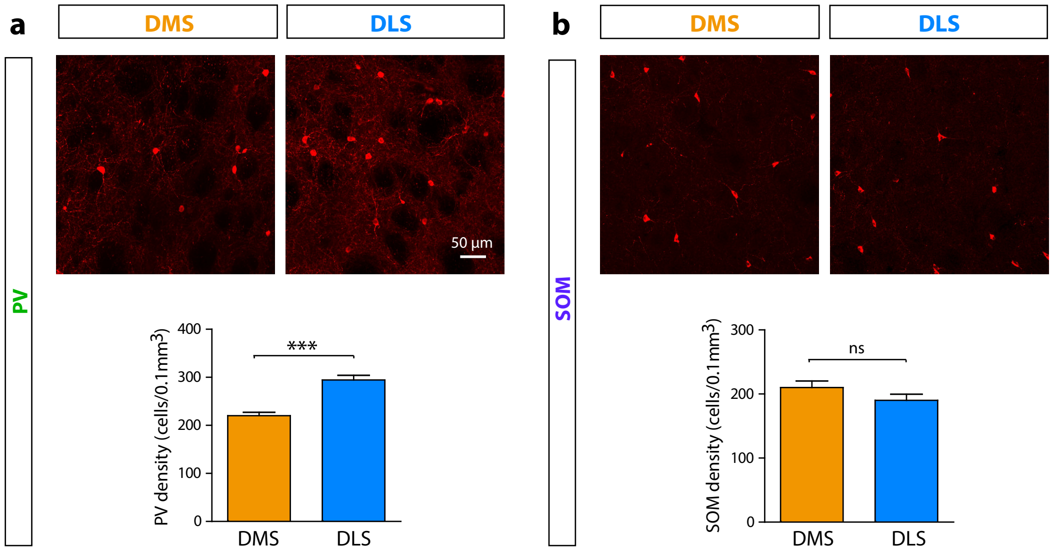

Supplementary Figure 8: Distribution of interneurons in adult animals

(**a**) Similarly to P30-P40 animals, adult (~6 month-old) animals also displayed a gradient in the distribution of PV cells. PV cells were particularly enriched in the DLS when compared to the DMS (+174%, 294.4±9.66 cells per 0.1mm3 in DLS vs 220.0±7.23 in DMS, p=0.0002, n=3 mice). (**b**) SOM cells were equally distributed in DMS and DLS (210.0±10.35 in DMS and 190.3±9.67 in DLS, p=0.1790, n=3 mice).

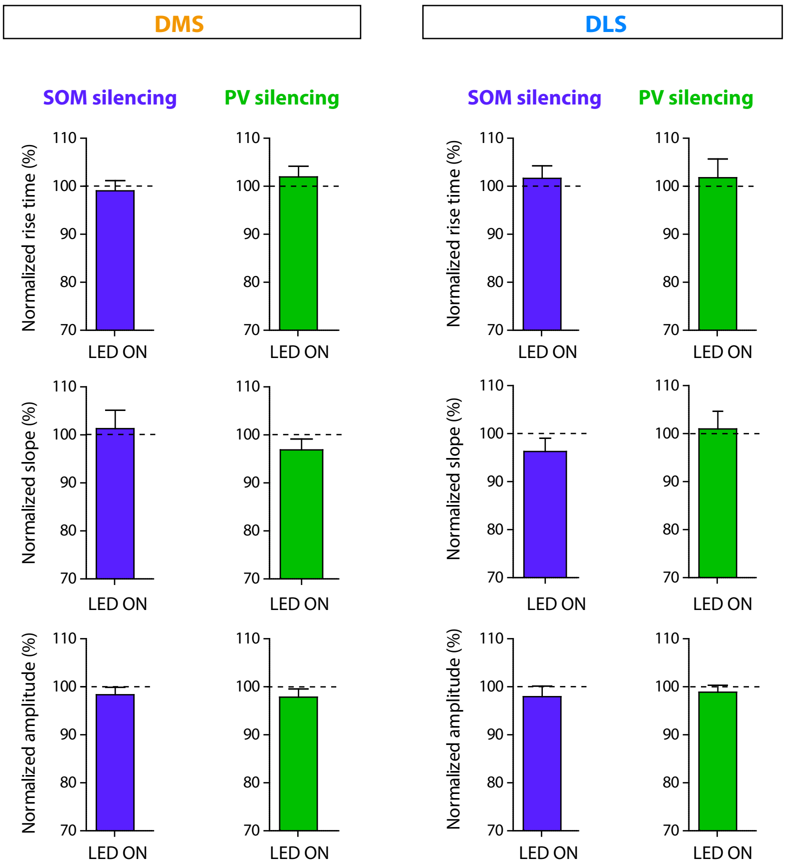

Supplementary Figure 9: Effect of PV and SOM cells silencing on cortically-evoked EPSPs in SPNs. Opto-inhibition of interneurons did not have any significant effect on rise time, slope and amplitude of EPSP. For rise time: p=0.2014 (n=16) for DMS-PV, p=0.3169 (n=20) for DMS-SOM, p=0.2070 (n=19) for DLS-PV and p=0.8672 (n=11) for DLS-SOM. For slope: p=0.0549 (n=16) for DMS-PV, p=0.5036 (n=20) for DMS-SOM, p=0.6676 (n=19) for DLS-PV and p=0.7781 (n=11) for DLS-SOM. For amplitude: p=0.0537(n=16) for DMS-PV, p=0.5871 (n=20) for DMS-SOM, p=0.3468 (n=19) for DLS-PV and p=0.5430 (n=11) for DLS-SOM (paired-test).

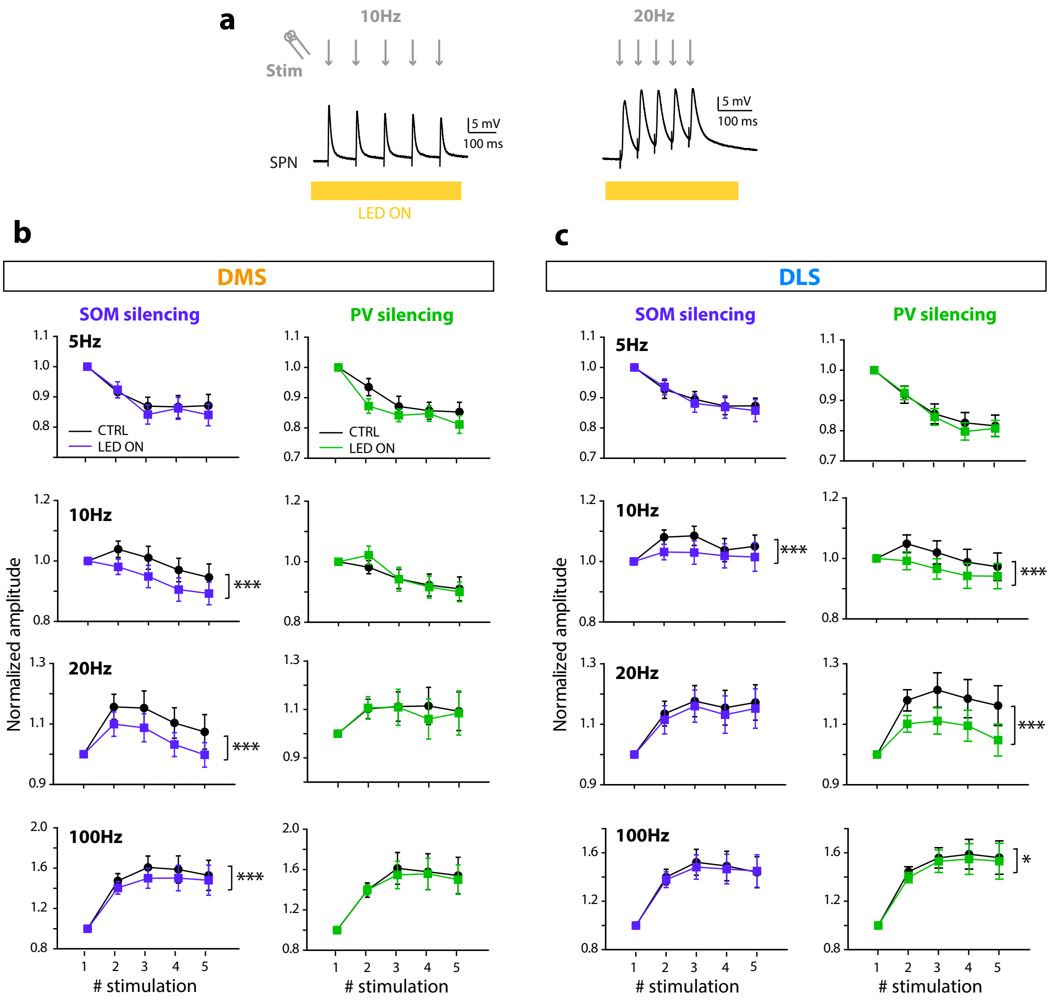

Supplementary Figure 10: SOM and PV interneurons favor activity summation of sustained cortical activity in SPNs at various frequencies.

(**a**) Representatives EPSPs recorded in SPNs in response to cortical stimulation trains at 10 Hz and 20 Hz. (**b**) In DMS, temporal summation of EPSPs in SPNs either in control conditions (black) or when SOM (purple) or PV cells (green) were inhibited. Opto-inhibition of SOM cells induced a significant decrease in of EPSP time course for trains at 10, 20 and 100 Hz ($F_{1,115}=2.17$, $p<0.0001$ for 10Hz, $F_{1,120}=1.59$, $p<0.0001$ for 20 Hz and $F_{1,60}=0.54$, $p<0.0001$ for 100 Hz, n=25, two-way Anova) while PV cell opto-inhibition had no effect onto EPSP time course except for 5Hz ($F_{1,50}=2.00$, $p=0.0195$ for 5Hz, $F_{1,70}=2.17$$p=0.03$ for 10 Hz, $F_{1,70}=0.06$, $p=0.1547$ for 20 Hz and $F_{1,30}=0.11$, $p=0.2402$ for 100 Hz, n=15). (**c**) In DLS, opto-inhibition of PV cells induced a significant decrease in summation of EPSPs for 10, 20 and 100 Hz induced trains ($F_{1,125}=1.17$, $p<0.0001$ for 10 Hz, $F_{1,125}=2.57$, $p<0.0001$ for 20 Hz and $F_{1,80}=0.11$ $p=0.0338$ for 100 Hz, n=26) while SOM cells silencing had no effect at 20 Hz and 100 Hz ($F_{1,80}=0.15$, $p=0.0542$ and $F_{1,60}=0.04$, $p=0.1536$ respectively, n=17) but had an effect at 10 Hz ($F_{1,80}=1.44$, $p<0.0001$, n=17). * $p<0.05$, ** $p<0.01$, *** $p<0.001$.

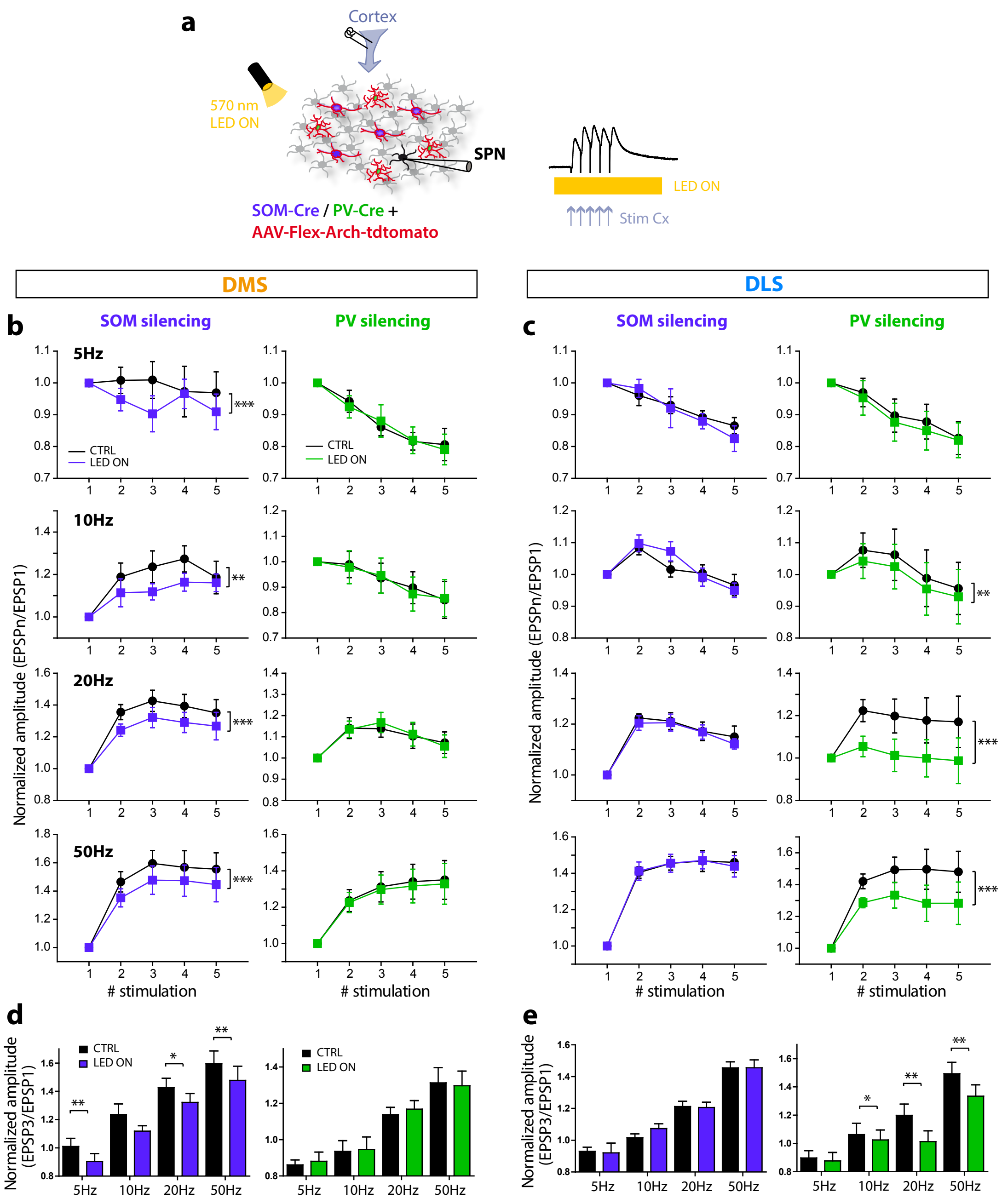

Supplementary Figure 11: SOM and PV interneurons favor activity summation of sustained cortical activity in SPNs after viral injections.

(**a**) After injections of AAVs in PV-Cre or SOM-Cre mice, cortically-evoked trains of EPSPs were recorded in SPNs in control conditions or during opto-inhibition of PV or SOM cells (ArchT, 570nm LED). (**b-c**) Temporal summations of EPSPs in SPNs after 5, 10, 20 and 50Hz cortical electrical stimulation either in control conditions (black) or when SOM (purple) or PV cells (green) were opto-inhibited. (**b**) In DMS, opto-inhibition of SOM cells induced a significant decrease in summation of EPSPs ($F_{1,30}=3.31$, $p<0.0001$ for 5Hz, $F_{1,25}=4.97$, $p<0.001$ for 10Hz, $F_{1,30}=3.93$, $p<0.0001$ for 20Hz, $F_{1,30}=2.06$, $p<0.0001$ for 50Hz, n=7 SPNs, Two-way Anova) whereas PV cell opto-inhibition had no effect ($F_{1,30}=0.01$, $p>0.05$ for 5Hz, $F_{1,30}=0.02$, $p>0.05$ for 10Hz, $F_{1,35}=0.02$, $p>0.05$ for 20Hz, $F_{1,35}=0.09$, $p<0.0001$ for 50Hz, n=8 SPNs). (**c**) In DLS, PV cells opto-inhibition induced a significant decrease in summation of EPSPs ($F_{1,25}=0.33$, $p>0.05$ for 5Hz, $F_{1,25}=7.49$, $p<0.001$, for 10Hz $F_{1,25}=12.91$, $p<0.0001$, for 20Hz $F_{1,25}=7.11$, $p<0.0001$, for 50Hz n=6 SPNs) and SOM cells opto-inhibition had no effect ($F_{1,25}=0.21$, $p>0.05$ for 5Hz, $F_{1,25}=0.37$, $p>0.05$ for 10Hz, $F_{1,25}=0.33$, $p>0.05$ for 20Hz, $F_{1,25}=0.01$, $p>0.05$ for 50Hz, n=6 SPNs). (d-e) Summary of the experiments of interneurons silencing on temporal summation: ratio of the 3rd EPSP compared to the first one for the different tested frequencies. (**d**) In DMS, there was no effect of PV cell opto-inhibition whereas SOM cell opto-inhibition strongly affects the summation of EPSPs in the trains for most activation frequencies (5, 20 and 50 Hz). (**e**) On the contrary, in DLS, PV cells strongly affected the summation of EPSPs in the trains for 10, 20 and 50 Hz while silencing of SOM cells had no effect.

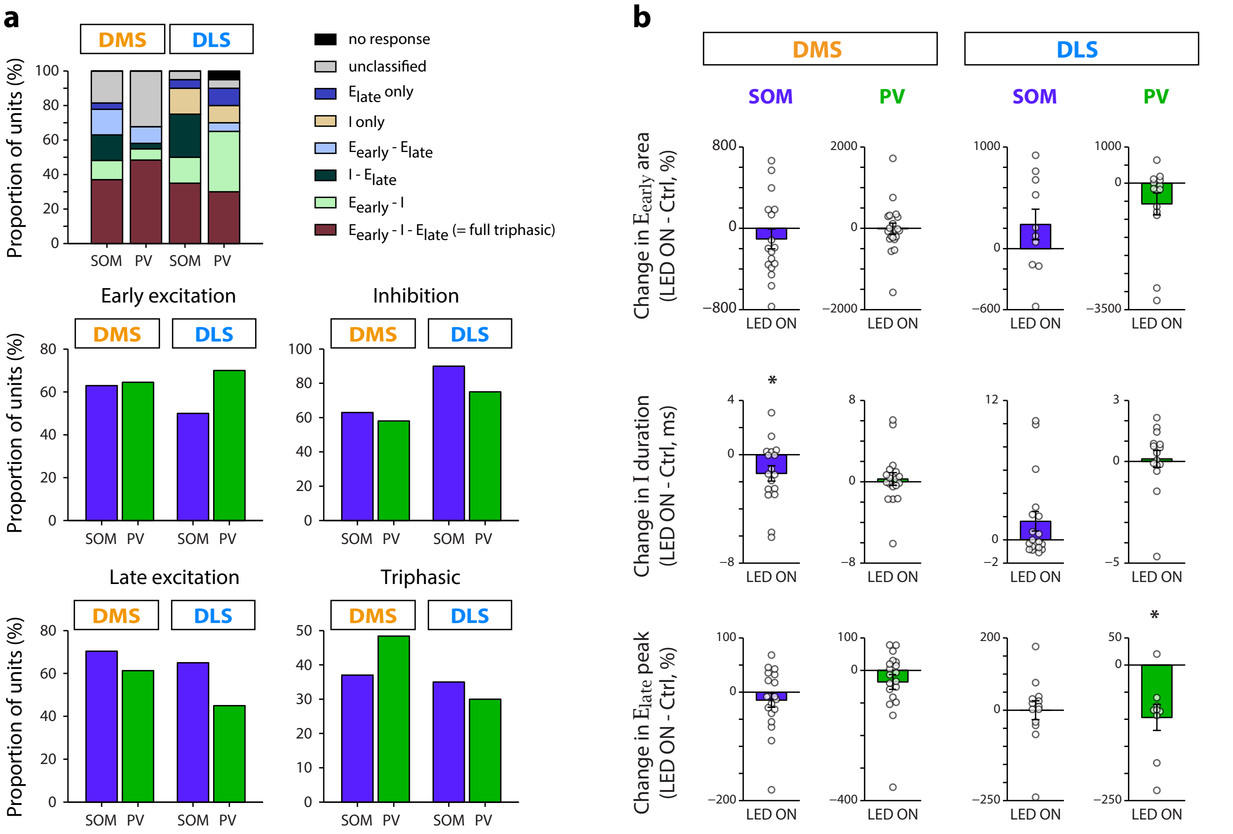

Supplementary Figure 12: Cortical stimulation evoked responses in SNr units.

(**a**) Patterns of cortical stimulation evoked responses were similar in all 4 conditions. Top: distributions of responses of all recorded SNr units to cortical stimulations in SOM-DMS (n=27 units), PV-DMS (n=31), SOM-DLS (n=20), and PV-DLS (n=20) conditions, classified by the presence or absence of any of the 3 "typical" phases: early excitation (Eearly), inhibition, and late excitation (Elate). Bottom: the proportion of units displaying each individual phase, or the full "triphasic" response were similar in all conditions (Fisher's exact test, Eearly: p=0.6297; Inhibition: p=0.07308; Elate: p=0.3583, Triphasic: p=0.5918). (**b**) Top: the area of the cortico-nigral early excitation phase is unchanged by opto-inhibition of striatal interneurons in all conditions. Middle: the duration of the cortico-nigral inhibition phase is significantly decreased when SOM interneurons are inhibited in the DMS (-1.38±0.57 ms, p=0.0274, n=17 units), but not in the 3 other conditions (PV-DMS: p=0.6674, n=18; SOM-DLS: p=0.0744, n=18; PV-DLS: p=0.7675, n=15). Bottom: the peak of the cortico-nigral late excitation phase is decreased by opto-inhibition of PV interneurons in DLS (-96.8±23.9%, p=0.0037, n=9 units), but not in the 3 other conditions (PV-DMS: p=0.1384, n=19; SOM-DMS: p=0.2732, n=19; SOM-DLS: p=0.9975, n=13).

	SPNs<br>n=140	PV cells<br>n=70	SOM cells<br>n=46	SPNs vs PVs	SPNs vs SOMs
**RMP** (mV)	-77,0±0,3	-70,5±0,4	-60,2±0,8	p<0,0001	p<0,0001
**Input resistance** (MΩ)	92,3±3,5	158,6±8,7	857,8±49,4	p<0,0001	p<0,0001
**Rheobase** (pA)	212,9±6,6	161,4±8,9	16,9±2,0	p<0,0001	p<0,0001
**Frequency** (Hz)	13,9±0,2	52,6±2,0	42,7±3,3	p<0,0001	p<0,0001

Supplementary Table 1: Basic membrane and spiking properties of PV and SOM interneurons compared to SPNs. All parameters were significantly different from interneurons to SPNs (unpaired t-test). RMP: resting membrane potential.

Phase of the SNr response		light	SOM-DMS	PV-DMS	SOM-DLS	PV-DLS
**Early Excitation**	*peak*	Off	173 ± 35 %	293 ± 50 %	312 ± 78 %	381 ± 108 %
(hyperdirect pathway)		On	173 ± 26 %	268 ± 47 %	348 ± 90 %	348 ± 97 %
		Δ	- 0.4 ± 13 %	- 25 ± 16 %	+ 36 ± 25 %	- 32 ± 30 %
SOM-DMS n=17	*onset*	Off	4.6 ± 0.8 ms	3.9 ± 0.4 ms	3.0 ± 0.2 ms	3.3 ± 0.4 ms
PV-DMS n=20		On	4.6 ± 0.7 ms	4.2 ± 0.7 ms	3.0 ± 0.2 ms	4.1 ± 0.8 ms
SOM-DLS n=10		Δ	+ 0.01 ± 0.7 ms	+ 0.3 ± 0.4 ms	+ 0.03 ± 0.2 ms	+ 0.8 ± 0.9 ms
PV-DLS n=14	*offset*	Off	15.7 ± 1.5 ms	14.2 ± 0.7 ms	12 ± 1.1 ms	12.1 ± 1.6 ms
		On	14.7 ± 1.2 ms	14.9 ± 0.8 ms	12.6 ± 1.0 ms	12.7 ± 1.9 ms
		Δ	- 1.0 ± 0.9 ms	+ 0.7 ± 0.4 ms	+ 0.7 ± 0.5 ms	+ 0.6 ± 0.9 ms
	duration	Off	11.1 ± 1.1 ms	10.3 ± 0.7 ms	9.0 ± 1.2 ms	8.8 ± 1.6 ms
		On	10.1 ± 0.8 ms	10.6 ± 0.9 ms	9.6 ± 1.0 ms	8.6 ± 1.6 ms
		Δ	- 1.0 ± 0.8 ms	+ 0.3 ± 0.6 ms	+ 0.6 ± 0.5 ms	- 0.2 ± 0.3 ms
	area	Off	1157 ± 246 %	1724 ± 340 %	1894 ± 593 %	2386 ± 842 %
		On	1053 ± 191 %	1714 ± 335 %	2132 ± 669 %	1811 ± 573 %
		Δ	- 105 ± 97 %	- 10 ± 139 %	+ 238 ± 150 %	- 575 ± 303 %
**Inhibition**	*peak*	Off	-89 ± 5 %	-95 ± 2 %	-88 ± 5 %	-95 ± 3 %
(direct pathway)		On	-84 ± 6 %	-91 ± 4 %	-87 ± 6 %	-96 ± 2 %
		Δ	+ 5 ± 3 %	+ 4 ± 4 %	+ 1.1 ± 2 %	- 0.3 ± 1 %
SOM-DMS n=17	*onset*	Off	13.3 ± 1.7 ms	13.1 ± 0.5 ms	8.8 ± 1.2 ms	12.3 ± 1.5 ms
PV-DMS n=18		On	13.5 ± 1.7 ms	13.9 ± 1.1 ms	8.8 ± 1.2 ms	12.3 ± 1.5 ms
SOM-DLS n=18		Δ	+ 0.2 ± 0.3 ms	+ 0.7 ± 0.8 ms	+ 0.02 ± 0.2 ms	- 0.02 ± .04 ms
PV-DLS n=15	*offset*	Off	28.6 ± 1.8 ms	24.8 ± 1.8 ms	31.8 ± 3.3 ms	36.8 ± 3.1 ms
		On	27.4 ± 1.5 ms	25.8 ± 2.0 ms	33.4 ± 3.2 ms	36.9 ± 3.1 ms
		Δ	- 1.2 ± 0.5 ms * (p=0.0304)	+ 1.0 ± 1.0 ms	+ 1.6 ± 0.9 ms	+ 0.1 ± 0.2 ms
	duration	Off	15.3 ± 1.9 ms	11.7 ± 1.8 ms	23.0 ± 3.2 ms	24.4 ± 2.7 ms
		On	13.9 ± 1.7 ms	12.0 ± 1.6 ms	24.6 ± 3.2 ms	24.6 ± 2.6 ms
		Δ	- 1.4 ± 0.6 ms * (p=0.0274)	+ 0.3 ± 0.6 ms	+ 1.6 ± 0.8 ms	+ 0.1 ± 0.4 ms
	area	Off	1038 ± 151 %	856 ± 153 %	1663 ± 275 %	1814 ± 211 %
		On	964 ± 142 %	881 ± 143 %	1710 ± 269 %	1860 ± 217 %
		Δ	- 74 ± 35 % * (p=0.0481)	24 ± 35 %	+ 47 ± 68 %	+ 47 ± 61 %
**Late Excitation**	*peak*	Off	254 ± 46 %	327 ± 49 %	252 ± 57 %	468 ± 108 %
(indirect pathway)		On	240 ± 40 %	292 ± 40 %	252 ± 55 %	372 ± 96 %
		Δ	- 15 ± 13 %	- 35 ± 23 %	- 0.1 ± 26 %	- 97 ± 24 % ** (p=0.0037)
SOM-DMS n=19	*onset*	Off	26.4 ± 1.8 ms	23.2 ± 1.0 ms	24.1 ± 2.2 ms	22.3 ± 2.5 ms
PV-DMS n=19		On	25.5 ± 1.5 ms	23.3 ± 1.1 ms	25.1 ± 2.4 ms	22.4 ± 2.4 ms
SOM-DLS n=13		Δ	- 0.9 ± 0.4 ms * (p=0.0207)	+ 0.1 ± 0.2 ms	+ 1.0 ± 0.5 ms	+ 0.05 ± 0.3 ms
PV-DLS n=9	*offset*	Off	42.0 ± 1.1 ms	38.2 ± 1.4 ms	41.6 ± 1.6 ms	37.6 ± 2.4 ms
		On	42.0 ± 1.1 ms	39.0 ± 1.3 ms	40.9 ± 1.9 ms	38.5 ± 2.6 ms
		Δ	+ 0.1 ± 0.8 ms	+ 0.8 ± 0.7 ms	- 0.7 ± 1.5 ms	+ 0.9 ± 1.2 ms
	duration	Off	15.5 ± 1.7 ms	15.0 ± 1.3 ms	17.5 ± 1.7 ms	15.3 ± 2.3 ms
		On	16.5 ± 1.8 ms	15.7 ± 1.4 ms	15.8 ± 1.5 ms	16.1 ± 2.0 ms
		Δ	+ 1.0 ± 0.7 ms	+ 0.7 ± 0.7 ms	- 1.7 ± 1.5 ms	+ 0.8 ± 1.3 ms
	area	Off	2025 ± 369 %	2437 ± 428 %	2196 ± 511 %	3931 ± 1002 %
		On	2104 ± 375 %	2246 ± 356 %	2123 ± 511 %	3072 ± 758 %
		Δ	+ 79 ± 86 %	- 191 ± 149 %	- 73 ± 181 %	-859 ± 308 % * (p=0.0237)

Supplementary Table 2: Opto-inhibition of striatal SOM or PV interneurons in DMS or DLS differentially affects properties of cortical-stimulation evoked triphasic response in SNr neurons. (mean ± sem).
